# A case study on *Salmonellaenterica* serovar Typhimurium at a dairy farm associated with massive sparrow death

**DOI:** 10.1186/s13028-016-0205-8

**Published:** 2016-04-26

**Authors:** Yukino Tamamura, Ikuo Uchida, Kiyoshi Tanaka, Yoshinori Nakano, Hidemasa Izumiya, Tatsufumi Takahashi, Naoya Kikuchi

**Affiliations:** 1 Hokkaido Research Station, National Institute of Animal Health, 4 Hitsujigaoka, Toyohira, Sapporo, 062-0045 Hokkado Japan; 2 Bacterial and Parasitic Diseases Research Division, National Institute of Animal Health, Tsukuba, 305-0856 Ibaraki Japan; 3 United Graduate School of Veterinary Sciences, Gifu University, 1-1 Yanagido, Gifu-Shi, 501-1193 Gifu Japan; 4 Department of Pathobiology, School of Veterinary Science, Rakuno Gakuen University, 582 Bunkyodai-midorimachi, Ebetsu, 069-8501 Hokkaido Japan; 5 Sorachi Livestock Hygiene Service Centre, 12-37 Okayama, Iwamizawa, 079-01 Hokkaido Japan; 6 Department of Bacteriology, National Institute of Infectious Diseases, 1-23-1 Toyama, Shinjuku-ku, 162-8640 Tokyo Japan

**Keywords:** *Salmonella* Typhimurium, Cattle, Sparrow, Japan, Pulsed-field gel electrophoresis, Multiple-locus variable-number tandem-repeat analysis

## Abstract

**Background:**

*Salmonella* *enterica* Typhimurium (*S*. Typhimurium) is the most common cause of bovine salmonellosis in Japan and where it is also cause of salmonellosis in wild birds. In 2008, a postpartum cow at a dairy farm developed diarrhea caused by *S*. Typhimurium. The herd was extensively surveilled for *Salmonella* sp. and we characterized bacterial isolates from this and other cows to determine the source of infection.

**Results:**

Eight isolates of *S*. Typhimurium from cattle were identified as phage type DT40 and showed a 100 % similarity by pulsed-field gel electrophoresis and the same or similar multiple-locus variable-number tandem-repeat analysis profiles as those of *S*. Typhimurium isolated from dead sparrows (*Passer montanus*) collected at Asahikawa in 2006. *S*. Typhimurium DT40 was considered to be a major cause of high sparrow mortality in Hokkaido in 2005–2006 and 2008–2009, suggesting that DT40 maintained in sparrows was transmitted to cattle.

**Conclusions:**

*S*. Typhimurium DT40 may be transmitted from sparrows to dairy cattle.

## Background

*Salmonella* infections are of great concern for both livestock and human health. *Salmonella* *enterica* serovar Typhimurium (*S*. Typhimurium) is the most common cause of bovine salmonellosis in Japan and the bacterium causes salmonellosis in wild birds as well. In 2005–2006, a large number of dead sparrows were observed in Hokkaido, Japan [1]. *S.* Typhimurium phage type DT40, which caused massive bird deaths in Europe [2–5], was identified as one causative factor in the 2005–2006 Japanese incident. In 2008–2009, another mass mortality of sparrows occurred and *S.* Typhimurium DT40 was isolated from dead birds [6].

In 2008, a postpartum cow in a dairy farm in central Hokkaido developed diarrhea caused by *S*. Typhimurium. We surveyed the extent of contamination and characterized bacterial isolates to help prevent disease transmission. Furthermore, we investigated the relationship between the bovine case of salmonellosis and the mass mortality of sparrows.

At the start of the study, the farm had 100 milking and dry cows, and 50 heifers and calves. No new cattle were introduced during the study period. Following the initial case of diarrhea on February 8, 2008, fecal samples were collected from all cattle and environmental samples were obtained from the aisles, neck rails, feed cups, and water cups in barns and pastures. Samples were collected every second week for 1 year, except in July and October 2008 and February 2009, when samples were collected once a month; samples were not collected in November and December 2008 (Table 1).

**Table 1:** Fecal and environmental culture results

Sampling date	Number of *Salmonella*-positive samples/total (%)			
	Lactating cows	Calves	Environment	Total
08 Feb 2008	1^a^			
12 Feb	0/90	0/67	6/42 (14 %)	6/199 (3.0 %)
27 Feb	1/89 (1.1 %)	1/66 (1.5 %)	0/41	2/196 (1.0 %)
08 Mar	0/96	0/69	0/47	0/212
18 Mar	0/89	0/70	0/46	0/205
03 Apr	1/88 (1.1 %)	0/71	5/135 (3.7 %)	6/294 (2.0 %)
17 Apr	0/83	0/66	0/51	0/200
07 May	0/84	0/58	0/50	0/192
21 May	0/117	0/33	0/50	0/200
4 Jun	0/110	0/40	0/50	0/200
18 Jun	0/108	0/39	0/50	0/197
16 Jul	0/106	0/43	0/50	0/199
11 Aug	1/90 (1.1 %)	0/54	0/50	1/194 (0.51 %)
14 Aug	N	N	0/23	0/23
1 Sep	0/24	N	N	0/24
16 Sep	0/85	0/63	0/56	0/204
22 Oct	0/89	0/57	0/56	0/202
13 Feb 2009	0/89	0/58	0/50	0/197
Total	3/1437 (0.20 %)	1/854 (0.11 %)	11/847 (1.3 %)	15/3138 (0.48 %)

*N* not done, *a* initial case

Samples were incubated in 10 ml of Hajna tetrathionate broth (Eiken, Tokyo) and subsequently subcultured on desoxycholate-hydrogen sulfide-lactose agar (Nissui, Tokyo). Colonies were confirmed as *Salmonella* using polyvalent antisera through serovar identification, performed using the slide agglutination method of Kaufmann and White [7]. The susceptibility of all the isolates to antimicrobial agents was determined by the disk diffusion test on Mueller–Hinton agar (Difco, Detroit, MI) according to the standards and interpretive criteria of the National Committee for Clinical Laboratory Standards [8]. The following antibiotic disks were used: ampicillin (10 μg), chloramphenicol (30 μg), streptomycin (10 μg), sulfonamides (250 μg), tetracycline (30 μg), kanamycin (30 μg), sulfamethoxazole and trimethoprim (23.75 and 1.25 μg, respectively), cefazolin (30 μg), ceftazidime (30 μg), cefotaxime (30 μg), nalidixic acid (30 μg), gentamicin (10 μg), and ciprofloxacin (5 μg). The antibiotic disks were sourced from Becton–Dickinson Microbiology Systems, Cockeysville, MD. The plasmid DNAs of all isolates were isolated using the method described by Kado and Liu [9].

We performed molecular analysis of nine representative isolates with reference strain IS18-33 [10] and three isolates derived from dead sparrows (*Passer montanus*) collected in 2006 at Asahikawa where the largest number of the dead sparrows were observed [1]. Pulsed-field gel electrophoresis (PFGE) analysis was performed following the standard protocol of PulseNet with the restriction enzyme *Xba*I (Takara Bio, Otsu) [11]. Multiple-locus variable-number tandem-repeat analysis (MLVA) was performed using primers (STTR3-F, STTR3-R, STTR5-F, STTR5-R, STTR6-F, STTR6-R, STTR9-F, STTR9-R, STTR10pl-F, and STTR10pl-R) to amplify 5 loci through PCR [12]. For DNA extraction, isolates were suspended and boiled for 5 min, immediately cooled on ice for 5 min, and centrifuged at 13,000×*g* and 4 °C for 5 min. Supernatants were used as the DNA template for polymerase chain reaction. Plasmid DNA was isolated by the alkaline lysis method [10]. Phage typing was performed according to previously described methods [13].

Sample culture results are summarized in Table 1. *S*. Typhimurium was isolated from two milking cows, one dry cow, one calf, and 11 environmental samples. In February 2008, the month when the first case was observed, six strains of *S*. Typhimurium were isolated from feed cups of the parturition pen, which housed the initially presenting cow, and two strains were cultured from one milking cow and one calf. In April 2008, one strain was cultured from a milking cow and five strains were cultured from the environment simultaneously, including one isolate from a feed cup and two isolates each from the aisles of the barn and waiting circle in a milking barn. Thereafter, *S*. Typhimurium was not isolated until the isolation from a dry cow in August. *S*. Typhimurium was not isolated again after August.

**Fig. 1:**
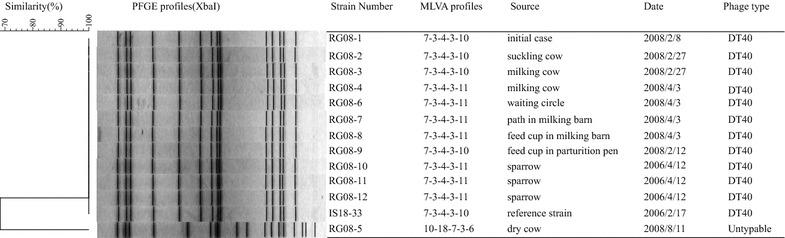
Dendrograms of *Xba*I pulsed-field gel electrophoresis (PFGE) and multiple-locus variable-number tandem-repeat (MLVA) profiles. PFGE and MLVA profiles were obtained for 13 *Salmonella* Typhimurium isolates. MLVA profiles are composed of 5 numbers indicating the repeat unit for each locus, with the following order: STTR3-STTR5-STTR6-STTR9-STTR10

We tested the antimicrobial susceptibility of isolated strains. All isolates were susceptible to all antimicrobials tested. Plasmids were extracted from all isolates. All isolates harbored 94-kb plasmids only. Representative isolates resulted in two different PFGE patterns (Fig. 1). Except for one isolate, which was obtained from a dry cow coded as 556 in August 2008 and named RG08-5, all isolates (including those from sparrows) showed PFGE patterns with a 100 % similarity. The PFGE profile of RG08-5 showed 68.3 % similarity with that of the other isolates.

Isolates were subjected to MLVA based on five variable tandem-repeat (VNTR) loci and characterized into three MLVA profiles (Fig. 1). Isolates RG08-1, -2, -3, and -9 showed the same profile, designated type A. Isolates RG08-4, -6, -7, and -8 showed another profile, designated type B, that differed from the type A profile at one VNTR location, STTR10. Isolate RG08-5 showed a profile designated type C, which differed from the other two profiles at three loci. The locus STTR10 is plasmid-borne and was previously reported to be hypervariable [14]. Profile B showed only one additional repeat at locus STTR10 compared to profile A and this difference presumably occurred during transmission, suggesting that the infection of *S.* Typhimurium in this farm may have been caused by a single clone until April 2008.

Only one cow showed clinical signs due to *S.* Typhimurium exposure. This cow was in the postpartum period and as cows are known to have compromised immunity around calving [15], this cow may have been particular susceptible. The isolated strain appears to be of low virulence to cattle.

RG08-5 showed the same PFGE profile and a similar MLVA profile to those of cluster VII-21, as previously described [10]. We cultured environmental samples including a portion of the cattle feed simultaneously with the isolation from the dry cow, but we failed to detect contamination by *S.* Typhimurium, indicating that the feed was probably not a source of infection for the dry cow. Furthermore, no new cattle were introduced in this farm during the study period. The dry cow infected with this isolate had fed in a pasture, where it may have acquired the infection from wild animals.

Except for isolate RG08-5, all isolates showed the same PFGE and MLVA profiles as those isolated from sparrows, indicating the possibility of transmission from sparrows to cattle. Bovine isolates displayed a weak catalase reaction, were negative for citrate utilization, and lacked the *sopE* gene, which has been associated with some epidemic *S*. Typhimurium strains in humans and animals [3]. These features are consistent with the *S*. Typhimurium DT40 strain isolated from dead infected wild birds [1, 3]. Actually, all isolates except RG08-5 were identified as DT40 (Fig. 1). Since DT40 is considered to be able to adapt to wild birds, sparrows were most likely source of the contamination.

Except for RG08-5, all isolates showed the same PFGE profile as the bovine isolate IS18-33, which was previously identified as belonging to PFGE cluster II-15 [10]. Furthermore, seven isolates of PFGE II-15 showed type A or B MLVA profiles [16]. These strains were isolated from cattle in central Hokkaido after 2006, indicating that isolates showing type A or B MLVA profiles had been disseminated in cattle in this area after a mass mortality event of sparrows. These isolates may also have been transmitted from sparrows to cattle.

The results indicate that *S.* Typhimurium strain DT40 causes not only large-scale death in birds, but also bovine salmonellosis. *S.* Typhimurium strain DT40 may be transmitted from sparrows to cattle by contamination of fed.
